# Ultrasensitive liquid sensor based on an embedded microchannel bulk acoustic wave resonator

**DOI:** 10.1038/s41378-024-00790-6

**Published:** 2024-10-11

**Authors:** Xiyu Gu, Yan Liu, Yuanhang Qu, Xiang Chen, Zesheng Liu, Yao Cai, Wenjuan Liu, Shishang Guo, Chengliang Sun

**Affiliations:** 1 Key Laboratory of Artificial Micro, and Nano-structures of Ministry of Education, School of Physics and Technology, Wuhan University, Wuhan, 430072 PR China; 2 School of Mathematical and Physical Sciences, Wuhan Textile University, Wuhan, 430200 PR China; 3 The Institute of Technological Sciences, Wuhan University, Wuhan, 430072 PR China; 4 School of Microelectronics, Wuhan University, Wuhan, 430072 PR China

**Keywords:** Electrical and electronic engineering, Sensors, Structural properties

## Abstract

The high-frequency and high-quality factor characteristics of bulk acoustic wave (BAW) resonators have significantly advanced their application in sensing technologies. In this work, a fluidic sensor based on a BAW resonator structure is fabricated and investigated. Embedded microchannels are formed beneath the active area of the BAW device without the need for external processes. As liquid flows through the microchannel, pressure is exerted on the upper wall (piezoelectric film) of the microchannel, which causes a shift in the resonant frequency. Using density functional theory, we revealed the intrinsic mechanism by which piezoelectric film deformation influences BAW resonator performance. Theoretically, the upwardly convex piezoelectric film caused by liquid flow can increase the resonant frequency. The experimental results obtained with ethanol solutions of different concentrations reveal that the sensor, which operates at a high resonant frequency of 2.225 GHz, achieves a remarkable sensitivity of 5.1 MHz/% (221 ppm/%), with an ultrahigh linearity of 0.995. This study reveals the intrinsic mechanism of liquid sensing based on BAW resonators, highlights the potential of AlN/Al_0.8_Sc_0.2_N composite film BAW resonators in liquid sensing applications and offers insights for future research and development in this field.

## Introduction

Owing to their excellent stability, resolution, and accuracy, as well as their low cost, compact size, and properties that allow devices to be easily interfaced with digital systems, resonators have attracted widespread interest in the detection and characterization of liquids. The ability to detect the concentration of liquids in a mixture is critical for many applications, such as water quality assessment^1^, chemical processing^2^, environmental monitoring^3^, and biosensing^4^. Resonator-based fluidic sensors monitor liquids through changes in the spectral response of the resonator, such as the quality factor (Q), resonance amplitude, and resonant frequency. Resonators commonly used as sensors can be divided into microwave resonators and piezoelectric resonators. Some studies have investigated split ring resonators (SRRs) and their derived resonator structures to realize high-sensitivity fluid sensors based on microwave resonators^5,6^. However, miniaturization, integration, anti-interference, and high sensitivity remain major challenges for fluid sensors in the detection and characterization of binary mixtures. The size of microwave resonators is larger than that of piezoelectric resonators. An increasing number of studies have investigated piezoelectric resonators to achieve compact size and high-sensitivity sensors based on piezoelectric resonators^7,8^.

Piezoelectric resonators are known for their precise control of frequency^9^. Bulk acoustic wave (BAW) resonators, as tiny piezoelectric devices manufactured by microelectromechanical systems (MEMS), have significant advantages as high-precision resonant sensors^10^, including smaller size, complementary metal oxide semiconductor (CMOS) compatibility, higher sensitivity, and low fabrication and measurement costs^11^. In addition, BAW resonators, as sensors that convert physical signals to electrical signals, have the advantage of high frequency, which can avoid interference. With the maturity of the BAW resonator manufacturing process, an increasing number of types of sensors based on BAW resonators have been designed. Using a BAW resonator, Yan et al. fabricated a mass sensor with a high sensitivity of 8–10 kHz cm^2^/ng at 3–4 GHz^12^. He et al. investigated the pressure sensing of a BAW resonator with a sensitivity of approximately −17.4 ppm kPa^−1, ^^13^.

BAW resonators also show great application potential in liquid sensors. Link et al. utilized a BAW resonator to detect water and 59% glycerol^14^. Vorobiev et al.^15^ and Rezazadeh et al.^16^ demonstrated the potential of the BAW resonator in characterizing the viscosity of liquids. Chen et al. fabricated a BAW resonator with c-axis tilted AlN films to achieve a sensitivity of 15 Hz cm^2^/μg for liquid loading^17^. In 2017, Liang et al. designed a glucose sensor with a sensitivity of 10.8 MHz/M through a glucose solution flowing on a BAW resonator^18^. Zhou et al. extended the application of BAW resonators in liquid viscosity sensing^19^. However, the intrinsic mechanism of resonant liquid sensors based on BAW resonators is lacking.

**Fig. 1:**
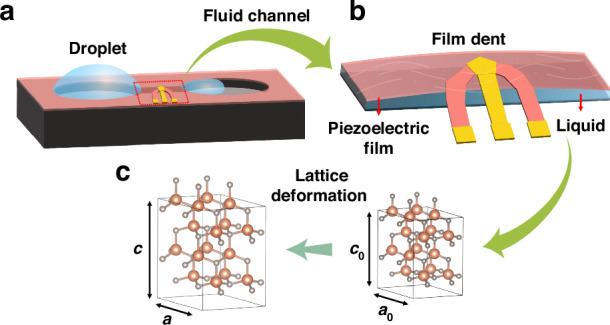
Mechanism of microchannel-type sensor. **a** Schematic of an embedded microchannel-type sensor, **b** schematic of the deformation of a piezoelectric film when liquid flows in a microchannel, and **c** schematic of wurtzite structure hexagonal supercell variation of AlN when liquid flows in a microchannel

In this work, we presented a high-sensitivity liquid sensor with an embedded microchannel under the AlN/AlScN BAW resonator, with the same fabrication process (6 masks) as the BAW resonator, as shown in Fig. 1a. Through the application of DFT calculations and finite element methods (FEMs), it was discovered that the frequency drift of the BAW resonator is caused by the deformation of the piezoelectric film, which is influenced by the liquid flow beneath the resonator, as shown in Fig. 1b, c. Different frequency drifts can reflect the concentration and type of liquid. These experimental results are in good agreement with our simulation results.

## Theory and design

Based on the frequency sensitivity of the BAW resonator, we designed embedded microchannels under BAW resonators for ultrasensitive sensing in liquids, as shown in Fig. 1a. This high-sensitivity liquid sensor has a low fabrication cost because it uses the same fabrication process (6 masks) as the BAW resonator. In this design, when liquid flows through the microchannel under the action of droplet pressure, the piezoelectric film under the microchannel will undergo mechanical deformation, as shown in Fig. 1b. The pressure formula of liquid flow on the microchannel wall is given by

1 $$P=\frac{8\mu {vl}}{{d}_{h}^{2}}$$

where *μ* is the fluid viscosity, *v* is the average fluid velocity, *l* is the unit flow length, and *d*_*h*_ is the hydraulic diameter of the microchannel. According to Formula (1), the deformation of the microchannel wall is directly influenced by the fluid viscosity.

**Fig. 2:**
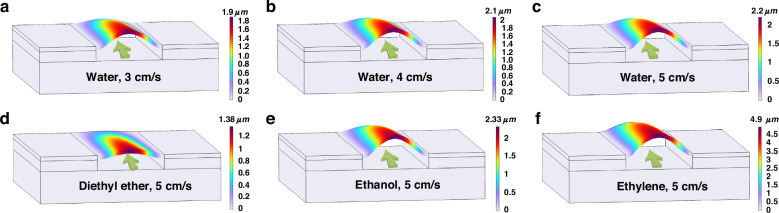
FEM simulation result of microchannel-type sensor. Deformation of piezoelectric films under microchannels under different liquid conditions: **a** 3 cm/s, water, **b** 4 cm/s, water, **c** 5 cm/s, water, **d** 5 cm/s, diethyl ether, **e** 5 cm/s, ethanol, and **f** 5 cm/s, ethylene glycol

The deformation of the piezoelectric film under the microchannel was investigated via the FEM when different liquids flow through the microchannel, as demonstrated in Fig. 2. As water with different velocities flows through the microchannel under the BAW resonator, the piezoelectric film deforms more as the flow speed increases, as depicted in Fig. 2a–c. Table 1 lists the peak displacement values of piezoelectric films under microchannels with various water velocities. When liquids with the same velocity are different, the deformations of the piezoelectric film are different. We select a variety of liquids (water, diethyl ether, ethanol, and ethylene glycol) to simulate the deformation of piezoelectric films under microchannels. Table 2 lists the viscosity parameters of these liquids at 25 °C. As shown in Fig. 2c–f, the deformation of the piezoelectric film increased as the viscosity of the liquid increased. Table 2 lists the peak displacement values of piezoelectric films under microchannels with various liquids flowing at 5 cm/s. These simulation results are consistent with the theoretical formula calculations. These different types of film deformation caused by different liquid flows directly affect the performance of the BAW resonator. These findings show that the embedded microchannels under BAW resonators have great application potential in ultrasensitive sensing in liquids.

**Table 1:** Deformation of piezoelectric films under microchannels with various water velocities (25 °C)

Velocity	3 cm/s	4 cm/s	5 cm/s
Peak displacement values	1.9 μm	2.1 μm	2.2 μm

**Table 2:** Deformation of piezoelectric films under microchannels with various liquids flowing at 5 cm/s (25 °C)

Liquid	Water	Diethyl ether	Ethanol	Ethylene glycol
Viscosity	0.91 × 10^−3^ Pa•s	0.224 × 10^−3^ Pa•s	1.074 × 10^−3^ Pa•s	1.61 × 10^−2^ PaPa•s
Peak displacement values	2.2 μm	1.38 μm	2.33 μm	4.9 μm

As the liquid flows through the microchannels under the resonator, the film bulges because the main effect of the z-direction is pressure on the film. This film deformation directly changes the lattice structure of the film material, which changes its physical properties. The mechanical and piezoelectric properties of piezoelectric materials are intrinsically linked to the resonant frequency and the effective electromechanical coupling coefficient of BAW resonators^20,21^. These characteristics significantly increase the potential of BAW resonators for sensing applications.

To analyze the effect of piezoelectric film bulging (the main effect of the z-direction exerting pressure) on the performance of the resonator, the change in the AlN lattice at different pressures in the z-direction is calculated. Through the first-principles software Material Studio (MS), which is based on density functional perturbation theory (DFT)^22^, a 32-atom hexagonal supercell of wurtzite AlN was constructed and used in calculations, as shown in Fig. 1c. The generalized gradient approximation of Perdew–Burke–Ernzerhof (GGA–PBE) was utilized to calculate the exchange-correlation total energy^23^. The first Brillouin-zone integrals are performed on a Monkhorst–Pack mesh of 9 × 9 × 3 k-points, and the plane wave cutoff is set to 570 eV. For precise calculation, the crystalline structures of 2 × 2 × 2 AlN used for calculation are a-axis lattice constants (*a*) and *c*-axis lattice constants (*c*), as depicted in Fig. 1c.

**Fig. 3:**
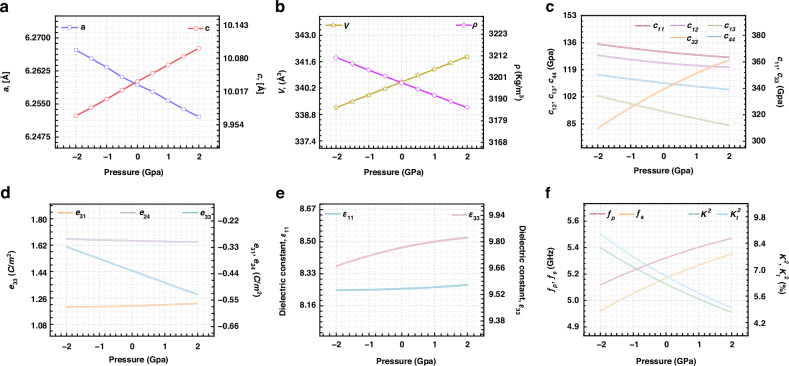
Theoretical performance variation of AlN-BAW resonator. **a** Lattice constants (*a* and *c*) of the AlN film dependence of the Z-direction exerted pressure; **b** lattice volume (*V*) and density ($\rho$) of the AlN film as a function of the z-direction pressure; **c** variation in the elastic constants of AlN in the residual z direction under pressure; **d** Piezoelectric coefficient (*e*_24_, *e*_31_, and *e*_33_) dependence of the z-direction exerted pressure. **e** Variations in the dielectric coefficients (${\varepsilon }_{11}$ and ${\varepsilon }_{33}$) of AlN in the z direction under pressure; **f** variation in important characterization parameters (*f*_*p*_, *f*_*s*_, *K*^2^, and ${K}_{t}^{2}$) of AlN-BAW resonators in the z direction under pressure

The pressure directly affects the *a*-axis and *c*-axis lattice constants of the film material^24^. To calculate the change in the AlN lattice at different pressures in the z direction, the linear response method was utilized with the Cambridge Sequential Total Energy Package (CASTEP)^22,23,25^. As presented in Fig. 3a, the *a* and *c* lattice constants had opposite linear relationships with the z-direction pressure in the range of −2 GPa to 2 GPa. The *a* lattice constant has a negative linear relationship with the z-direction pressure, and the *c* lattice constant has a positive linear relationship with the z-direction pressure. This law is consistent with the deformation of some materials under z-direction pressure^26^.

Moreover, as depicted in Fig. 3b, the lattice volume (*V*) also has a positive linear relationship with the z-direction pressure, and the lattice density ($\rho$) has a negative linear relationship with the z-direction pressure. These lattice changes directly affect physical properties, such as piezoelectric properties and the elastic stiffness tensor.

To obtain the elastic constants under different pressures in the z-direction, crystalline structures deformed at different pressures were computed via CASTEP. The variation in the relative elastic constants with pressure (*x*) can also be found in the calculation results, as shown in Fig. 3c. In the range of −2 GPa to 2 GPa, this trend was fitted by quadratic Eqs. (2)–(6):

2 $${c}_{11}\left(x\right)=0.2388{x}^{2}-2.4968x+367.542$$

3 $${c}_{12}\left(x\right)=0.2744{x}^{2}-1.8903x+123.237$$

4 $${c}_{13}\left(x\right)=0.1425{x}^{2}-4.6558x+92.858$$

5 $${c}_{33}\left(x\right)=-0.9146{x}^{2}+12.9104x+339.25$$

6 $${c}_{44}\left(x\right)=0.1391{x}^{2}-2.3071x+110.687$$

For the piezoelectric film used in BAW resonators, another crucial characteristic is the piezoelectric constant. Piezoelectricity is intimately linked to material polarization. In the absence of external fields, the overall macroscopic polarization (*P*) in a piezoelectric material consists of two components: strain-induced or piezoelectric polarization ($\delta P$) and spontaneous polarization (*P*^*s*^). The piezoelectric polarization is defined as^27^

7 $$\delta {P}_{i}=\sum _{j}{e}_{{ij}}{s}_{j}$$

where *e* is the piezoelectric stress coefficient. Since precision BAW resonators mainly use *e*_33_ to create vibrations along the z-axis, in this work, we only examine polarizations along the (0001) axis. In the c-axis orientation of AlN, the piezoelectric polarization can be simply expressed as^28^

8 $$\delta {P}_{3}={e}_{33}{s}_{3}+{e}_{31}({s}_{1}+{s}_{2})$$

where ${s}_{1}={s}_{2}=({\rm{a}}-{a}_{0})/{a}_{0}$ is the in-plane strain and ${s}_{3}=({\rm{c}}-{c}_{0})/{c}_{0}$ is the strain along the c-axis. *c*_0_ and *a*_0_ are the lattice constants in the equilibrium structure. Equation (8) is a macroscopic phenomenological equation. From a microscopic perspective, we separate the clamped ion term and the internal relaxation term^29^:

9 $${e}_{33}={e}_{33}^{(0)}+\left.\frac{\delta {P}_{3}}{\delta u}\right|_{{s}_{3}}\frac{{du}}{d{s}_{3}},$$

10 $${e}_{31}={e}_{31}^{(0)}+\left.\frac{\delta {P}_{3}}{\delta u}\right|_{{s}_{1}}\frac{{du}}{d{s}_{1}},$$

where

11 $${e}_{33}^{(0)}=\left.\frac{\delta {P}_{3}}{\delta {s}_{3}}\right|_{u}={c}_{0}\frac{\partial {P}_{3}}{\partial c},$$

12 $${e}_{31}^{(0)}=\left.\frac{\delta {P}_{3}}{\delta {s}_{1}}\right|_{u}=\frac{{a}_{0}}{2}\frac{\partial {P}_{3}}{\partial a},$$

The internal parameter *u* is defined as the bond length between anions and cations along the c-axis, and it determines the relative positions of atoms within the cell. Polarization changes caused linearly by relative sublattice shifts (variations in *u*) can be measured by the effective charge Z^*^:

13 $${Z}^{* }=\frac{\sqrt{3}{a}_{0}^{2}}{4e}\left.\frac{\delta {P}_{3}}{\delta u}\right|_{{s}_{3}}$$

where *e* is the electronic charge. According to formulas (9)–(13), the piezoelectric coefficients and the lattice constants are closely related.

Using the Vienna Ab initio Simulation Package (VASP), a set of piezoelectric coefficients was calculated for AlN lattices deformed under various pressures. After the influence of pressure applied in the z-direction on the piezoelectric coefficient is analyzed, their relationship can be approximated via a quadratic equation, as illustrated in Fig. 3d. The corresponding equations are fitted as follows:

14 $${e}_{33}\left(x\right)=5.714\times {10}^{-5}{x}^{2}-0.0816x+1.446$$

15 $${e}_{31}\left(x\right)=8.857\times {10}^{-4}{x}^{2}+0.0037x-0.574$$

16 $${e}_{15}\left(x\right)={e}_{24}\left(x\right)=3.57\times {10}^{-4}{x}^{2}-0.00318x-0.303$$

Additionally, the effect of pressure applied in the z-direction on the dielectric permittivity is investigated, as shown in Fig. 3e. Within the pressure range of −2 GPa to 2 GPa, the variation in relative permittivity with pressure (*x*) in the z-direction is described by quadratic Eqs. (17) and (18) in the following form:

17 $${\varepsilon }_{11}\left(x\right)=1.57\times {10}^{-3}{x}^{2}+0.0067x+8.25$$

18 $${\varepsilon }_{33}\left(x\right)=-1.01\times {10}^{-2}{x}^{2}+0.045x+9.77$$

These physical parameter formulas directly reflect the effect of pressure applied in the z-direction on the performance of piezoelectric devices, such as BAW resonators and filters. For MEMS BAW resonators, the frequency and bandwidth are extremely tightly controlled. The frequency and bandwidth of BAW resonators are limited by the elastic constant (*c*_33_), dielectric permittivity (${\varepsilon }_{33}$), and piezoelectric coefficient (*e*_33_)^30^. The corresponding formula is as follows^31^:

19 $$Z=\frac{1}{{jw}{C}_{0}}\left(1-{K}_{t}^{2}\frac{{kd}/2}{\tan ({kd}/2)}\right)$$

where *Z* is the input impedance; *w* is the angular frequency; *C*_0_ is the electrostatic capacitance; *d* is the film thickness of the device; ${K}_{t}^{2}$ is the piezoelectric coupling constant of the transversely clamped material (${K}_{t}^{2}={e}^{2}/({c}^{E}+\frac{{e}^{2}}{{\varepsilon }^{s}}){\varepsilon }^{s}={K}^{2}/{K}^{2}+1$, where ${K}^{2}={e}^{2}/{{c}^{E}\varepsilon }^{s}$ is the electromechanical coupling factor^32^); and *k* is the wavenumber. According to the variations in *c* and *e* with pressure, the variations in ${K}_{t}^{2}$ and *K*^2^ of AlN with pressure were calculated. They increase with increasing pressure, as shown in Fig. 3f. Since infinite input impedance corresponds to the parallel resonance of the lossless circuit, the corresponding acoustic frequency (*f*_p_) is given by

20 $${f}_{p}=(2n+1)\frac{{v}^{D}}{2d},n=\mathrm{0,1,2},\ldots$$

where ${v}^{D}=\sqrt{{c}^{E}/\rho ({K}^{2}+1)}$ is the acoustic velocity in the piezoelectric medium. The series resonant frequency (*f*_*s*_) is similarly obtained from Eq. (19) for *Z* = 0 and is therefore obtained from

21 $${K}_{t}^{2}=\frac{\frac{\pi }{2}\frac{{f}_{s}}{{f}_{p}}}{\tan \left(\frac{\pi }{2}\frac{{f}_{s}}{{f}_{p}}\right)}\approx \frac{{\pi }^{2}}{4}\frac{{f}_{s}}{{f}_{p}}\frac{({f}_{p}-{f}_{s})}{{f}_{p}}$$

In this sensor, which is based on BAW resonators, the first resonator frequency is used; therefore, *n* is equal to 0. Based on the 1 μm thick AlN film and the above equations, the ideal variations in *f*_*p*_ and *f*_*s*_ with pressure applied in the z direction can be obtained, as shown in Fig. 3f. They increase with increasing pressure applied in the z-direction, and the increasing trend of *f*_*s*_ is greater than that of *f*_*p*_, which is in agreement with the experimental study of Zhou et al.^19^.

## Results and discussion

### Film Fabrication and Characterization

**Fig. 4:**
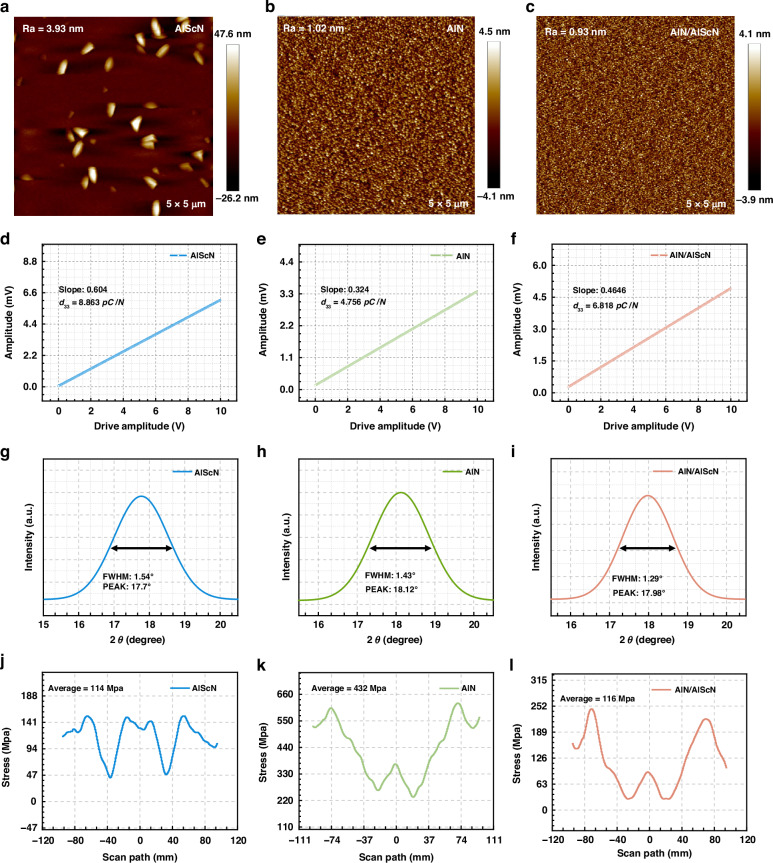
AlN/Al_0.8_Sc_0.2_N composite film characterization. AFM images of the **a** Al_0.8_Sc_0.2_N film, **b** AlN film, and **c** AlN/Al_0.8_Sc_0.2_N composite film. Piezoelectric amplitude curves of the **d** Al_0.8_Sc_0.2_N film, **e** AlN film, and **f** AlN/Al_0.8_Sc_0.2_N composite film. X-ray diffraction (XRD) rocking curves of the **g** Al_0.8_Sc_0.2_N film, **h** AlN film, and **i** AlN/Al_0.8_Sc_0.2_N composite film. Film-stress measurement curves of the **j** Al_0.8_Sc_0.2_N film, **k** AlN film, and **l** AlN/Al_0.8_Sc_0.2_N composite film

The piezoelectric properties of materials strongly influence the mechanical quality factors (*Q*_*M*_), *f*_*p*_, and ${K}_{t}^{2}$ of piezoelectric resonators^33^. Wei Wu et al. reported that the $-\Delta f/f$ of piezoelectric plate sensors increased with increasing electromechanical coupling constant *k*_31_ due to the extrinsic effect of polarization orientation switching at room temperature^34^. A. Talbi et al. also indicated that a better ${K}_{t}^{2}$ of the resonator could increase the sensitivity of the resonant sensor^35^. AlScN, which enhances the piezoelectric property of AlN, has been used to fabricate resonant sensors that are more sensitive than those based on AlN^36^. However, during the deposition of the AlScN film by the RF magnetron sputtering system, there are numerous abnormally oriented grains (AOGs). As demonstrated in Fig. 4a, atom force microscopy (AFM) revealed that the AOGs were large triangular pyramidal grains randomly distributed on the surface of the Al_0.8_Sc_0.2_N film. AOGs affect the piezoelectric and mechanical properties of AlScN films^37^.

Therefore, this work utilized an AlN/Al_0.8_Sc_0.2_N composite film (500 nm AlN + 500 nm Al_0.8_Sc_0.2_N) to inhibit the growth of AOGs, which was investigated in our previous research^38^. A 1 μm AlN, 1 μm Al_0.8_Sc_0.2_N, and 1 μm AlN/Al_0.8_Sc_0.2_N composite film (500 nm AlN + 500 nm Al_0.8_Sc_0.2_N) was prepared through an RF magnetron sputtering system. During deposition, the distance between the target and the substrate was 50 mm, and the deposition temperature was 200 °C. According to the AFM characterization results, no AOGs exist on the AlN/Al_0.8_Sc_0.2_N composite film, and the AlN/Al_0.8_Sc_0.2_N composite film has a relatively small root-mean-square (rms) roughness (AlScN: 3.93 nm; AlN: 1.02 nm; AlN/Al_0.8_Sc_0.2_N: 0.93 nm), as depicted in Fig. 4a–c. This smooth film surface reduces scattering losses, thereby improving the Q value of the resonator^39^. Through piezoresponse force microscopy (PFM), the piezoelectric properties (*d*_33_) of the AlN film, the Al_0.8_Sc_0.2_N film, and the AlN/Al_0.8_Sc_0.2_N composite film were measured, as shown in Fig. 4d-f. It is reasonable that *d*_33_ = 6.818 *pC*/*N* of the AlN/Al_0.8_Sc_0.2_N composite film is in the middle position among them. Moreover, the AlN/Al_0.8_Sc_0.2_N composite film improved the film quality of the piezoelectric layer in the BAW resonators. The full width at half maximum (FWHM) of the X-ray diffraction (XRD) rocking curves was used to characterize the film quality of these three piezoelectric films. As shown in Fig. 4g, h, and i), the FWHMs of the AlN film, Al_0.8_Sc_0.2_N film, and AlN/Al_0.8_Sc_0.2_N film were 1.43°, 1.54°, and 1.29°, respectively, which confirmed that the AlN/Al_0.8_Sc_0.2_N composite film had better crystal quality. In addition, the AlN/Al_0.8_Sc_0.2_N composite film neutralized the residual stress from the AlN film and the Al_0.8_Sc_0.2_N film, as demonstrated in Fig. 4j–l. An AlN/Al_0.8_Sc_0.2_N composite film could be an alternative for regulating the residual stress of a film to reduce the damage caused by residual stress in MEMS devices.

### Fabrication and experiments

**Fig. 5:**
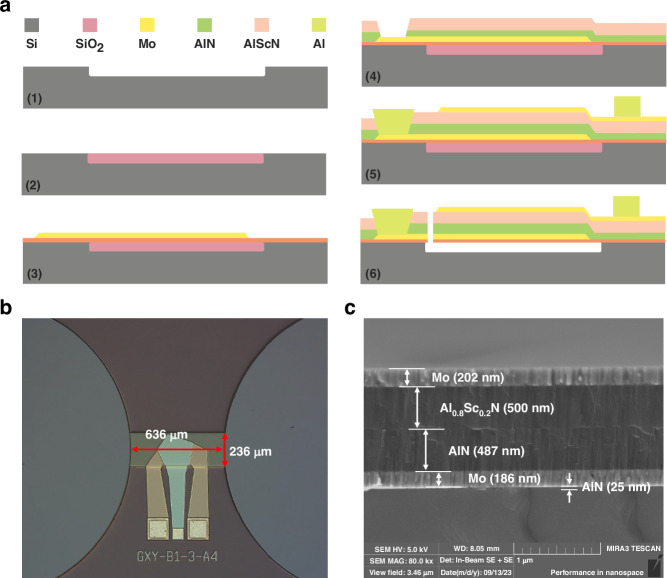
Main fabrication process and device characterization. **a** Main fabrication process steps for liquid sensors based on embedded microchannel BAW resonators; **b** optical microscopy (OM) image of the fabricated liquid sensor; and **c** SEM image of the AlN/Al_0.8_Sc_0.2_N composite film-based liquid sensor

Based on this sensor design, the fabrication process is illustrated in Fig. 5a. The high-sensitivity liquid sensor decreased the fabrication cost because the same fabrication process (6 masks) was used for the BAW resonators. (1) A 2.5 μm microchannel pool was etched by deep reactive ion etching (DRIE). (2) Then, the 3.5 μm released SiO_2_ layer was deposited via plasma-enhanced chemical vapor deposition (PECVD), and the excess SiO_2_ beside the swimming pool was removed through chemical mechanical polishing (CMP). (3) An RF magnetron sputtering system was used to deposit a 25 nm AlN seed film and a 200 nm Mo film. The Mo film is subsequently patterned and etched as bottom electrodes. (4) AlN and Al_0.8_Sc_0.2_N piezoelectric films were deposited successively. Inductively coupled plasma (ICP) was used to etch the AlN/Al_0.8_Sc_0.2_N composite film to expose the pads of the bottom electrodes. (5) The top Mo film was deposited and patterned as top electrodes. The Al film was evaporated and then peeled off into the pad shape. (6) The AlN/Al_0.8_Sc_0.2_N composite film was etched to form a release hole, and SiO_2_ in the microchannel pool and liquid hole was released via VHF. The embedded microchannel sensor under the BAW resonators was fabricated as shown in Fig. 5b. A scanning electron microscope (SEM) image of the cross-sectional BAW resonator is shown in Fig. 5c.

**Fig. 6:**
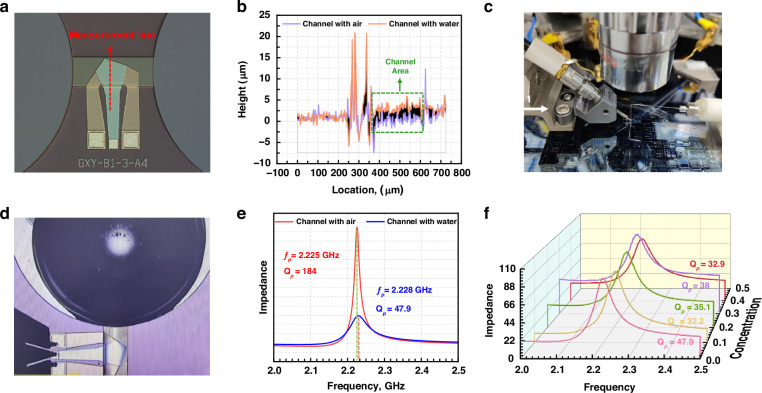
Experimental results of microchannel-type sensor. **a** Line of the height of the AlN/Al_0.8_Sc_0.2_N composite film; **b** height variation of the red line (**a**) before and after the liquid flows through the microchannel; **c** image of the setup of the liquid flow measurement; **d** measured behavior for liquid flow; **e** impedance signals measured as microchannel-filled air and water; and **f** impedance signals measured for different concentrations of microchannel-filled ethanol

Before testing, the height variation of the AlN/Al_0.8_Sc_0.2_N composite film along the red line was measured via confocal laser scanning microscopy (CLSM), as shown in Fig. 6a, b. This film height variation could reflect the film deformation before and after the liquid flows through the microchannel. When liquid water flows through it, the film on the microchannel bulges due to upward pressure, as demonstrated in Fig. 6b. These measurement results are in agreement with the theoretical calculations and simulation results.

The liquid flow impacts on the BAW resonator were measured via the setup illustrated in Fig. 6c. This setup pushed a fixed amount of liquid through the spiral annotation tube to drop into the liquid hole. The droplet pressure drives the solution to flow through the microchannel, as shown in Fig. 6d. First, the BAW resonator was measured in air by a Keysight network analyzer (N5222B) with a Cascade Microtech GSG probe station (Cascade, USA). This impedance signal measured as microchannel-filled air is depicted in Fig. 6e. While the GSG probe remained tested, we pushed water into the liquid hole. Therefore, the impedance signal can eliminate the influence of other interference. As shown in Fig. 6e, *f*_*p*_ increased at 3 MHz, and *Q*_*p*_ decreased sharply from 184 to 47.9 as water flowed. This reduction in *Q*_*p*_ occurred because the liquid hindered the vibration of the BAW resonator and increased the energy loss of the BAW resonator. This frequency draft originating from the bulged AlN/Al_0.8_Sc_0.2_N composite film confirms the accuracy of the first-principles calculation.

**Fig. 7:**
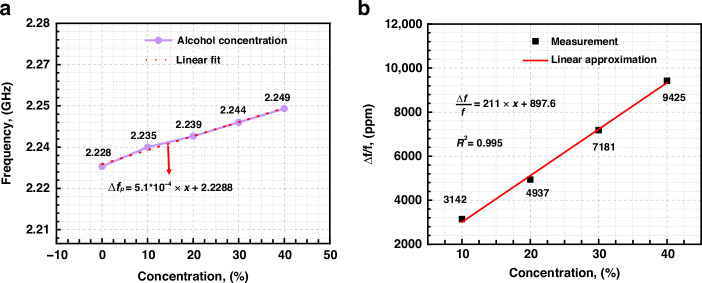
Sensitivity of microchannel-type sensor. **a** Frequency variation under different concentrations of microchannel-filled ethanol and **b** sensitivity ($\Delta f$/${f}_{p}$) of this microchannel liquid sensor

Subsequently, different concentrations of ethanol solutions were used to measure the sensitivity of this sensor. Figure 6f shows the impedance variations of the BAW resonator at different ethanol mass percentage concentrations (10%, 20%, 30%, and 40%). A noticeable frequency shift is observed with increasing ethanol concentration, and the quality factor (*Q*_*p*_) fluctuates with increasing ethanol concentration. Figure 7a shows the variation in *f*_*p*_ as a function of the ethanol concentration. After linear approximation, the measured frequency variation under ethanol concentration was 0.51 MHz/%, and the corresponding concentration sensitivity ($\Delta f$/${f}_{p}$) was 211 ppm/%, with an ultrahigh linearity of 0.995, as illustrated in Fig. 7b.

## Conclusions

Based on the pressure of liquid flow on the microchannel wall, this work designed and fabricated a high-sensitivity resonant liquid sensor with an embedded microchannel under BAW resonators. Through FEM simulations, it was determined that at the same flow rate, different viscosities of liquids caused different pressures on the microchannel wall. As the viscosity of the liquid increased, the deformation of the membrane wall also increased. When the liquid flows in the microchannel, the main force that causes the film to bulge is the z-direction pressure. Through first-principles calculations, we characterized the physical properties of the 32-atom hexagonal supercell of wurtzite AlN under different pressures in the z direction. These calculation results reveal the variation rule of the lattice constant, density, elastic constant, piezoelectric constant, etc. Subsequently, we revealed the intrinsic mechanism of how the z-direction pressure affects the performance of AlN-based BAW resonators. Theoretically, the upwardly convexness of the piezoelectric film caused by liquid flow can increase the resonator frequency.

During fabrication, the liquid sensor used an AlN/Al_0.8_Sc_0.2_N composite film to effectively suppress the abnormal orientation of the AlScN grains. The experimental results with different concentrations of ethanol reveal that our sensor, operating at a high resonant frequency of 2.225 GHz, achieves a remarkable sensitivity of 5.1 MHz/% (221 ppm/%) with an ultrahigh linearity of 0.995. These results of the frequency shift trend of the resonator are in agreement with the theoretical prediction. This study reveals the intrinsic mechanism of force sensing based on BAW resonators and offers insights for future research and development in this field.
